# *Oulema septentrionis* and *O. erichsonii* are neither conspecific nor melanic variants of *O. melanopus* as assessed by micro CT analysis of their lectotypes (Insecta, Coleoptera, Chrysomelidae, Criocerinae)

**DOI:** 10.3897/zookeys.720.19760

**Published:** 2017-12-11

**Authors:** Michael Schmitt, Gabriele Uhl

**Affiliations:** 1 Ernst-Moriz-Arndt-Universität, Allgemeine & Systematische Zoologie, Loitzer Str. 26, 17489 Greifswald, Germany

**Keywords:** Taxonomy, lectotype designation, 3D-reconstruction, aedeagus, flagellum, morphology

## Abstract

The investigation of the type series of *Oulema septentrionis* (Weise, 1880) and *Oulema erichsonii* (Suffrian, 1841) using Micro-computed X-ray tomography (µCT) revealed that neither species is a melanic variant of *Oulema melanopus* (Linnaeus, 1758) as has been suggested previously. Lectotypes of *Oulema septentrionis* (Weise, 1880) and *Oulema erichsonii* (Suffrian, 1841) are designated based on the study of type material. The male genitalia of *O. septentrionis* and *O. erichsonii* differ to an extent in the shape of the median lobe and flagellum that their status as separate species is – cautiously – confirmed by the present study.

## Introduction

In the western Palearctic there are *Oulema* species with a red and with a blue pronotum. The discussion as to how many species we should accept is ongoing. In catalogues (e.g. Schmitt 2010) and identification keys (e.g. Kippenberg 1994) five species with blue pronotum are listed: *O. septentrionis* (Weise, 1880), *O. erichsonii* (Suffrian, 1841), *O. obscura* (Stephens, 1831) = *O. gallaeciana* (Heyden, 1870) see Cox (2000), and *O. tristis* (Herbst, 1786). *O. septentrionis* is sometimes regarded a subspecies or even variety of *O. erichsonii* (e.g. Mohr 1966). A fifth species with a blue pronotum was described in 1964 from Italy, *O. maggistrettiorum* Ruffo, 1964. A recent review of the species with red pronotum (Bezdek and Baselga 2015) considered five different species occurring in Europe: *O. melanopus* (Linnaeus, 1758), *O. duftschmidi* (Redtenbacher, 1874), *O. mauroi* Bezdek & Baselga, 2015, *O. rufocyanea* (Suffrian, 1847) and *O. verae* Bezdek & Baselga, 2015. A sixth species, *O. hoffmannseggii* (Lacordaire, 1845), is listed by e.g., Warchałowski (2003, 2010).

On the website of the NERC- Centre for Ecology & Hydrology the hypothesis was published that specimens identified as *Oulema septentrionis* in Ireland could actually be melanic forms of *O. melanopus*: “The taxonomic status of *O. septentrionis* in Ireland is currently under review as there is evidence from dissections of the aedeagal flagellum that specimens from Ireland and Normandy are a melanic form of *O. melanopus*. The final outcome of this work is awaiting publication” (http://www.coleoptera.org.uk/species/oulema-septentrionis, last accessed 11.05.2017). We examined the type specimens of *O. septentrionis* and *O. erichsonii* non-distructively under microCT in order to inspect the median lobe and flagellum of the aedeagus and compare them with the those in the red-necked *Oulema* species *O. melanopus* and *O. duftschmidi* - as these two latter can hardly be separated by external morphological characters. With this investigation we attempt to assess two hypotheses: first, that *O. septentrionis* is a melanic form of *O. melanopus* and second that *O. septentrionis* and *O. erichsonii* are conspecific. The latter assessment is based on a morphological species concept (“morphospecies”). As long as there is no sound information at hand as to possible cross-breeding (“biospecies”) and ecological relationships (“ecospecies”), we use morphospecies as a proxy for bio- and ecospecies.

## Material and methods

From the collection of the Museum für Naturkunde Berlin (ZMUH) we received the syntype series of *Oulema septentrionis* (Weise, 1880), consisting of 11 specimens. From the collection of the Martin-Luther Universität Halle (MLUH) we received a syntype series of 14 specimens for *Oulema erichsonii* (Suffrian, 1841). Three syntype specimens of *Oulema septentrionis* and one syntype specimen of *Oulema erichsonii* were microCT-scanned with an Xradia Micro XCT-200 (Carl Zeiss X-ray Microscopy Inc.), using the 4× object lens units, at 40 kV and 8 W, with a pixel size of 5.36 µm. Tomography projections were reconstructed using the reconstruction software provided by XRadia. Volume rendering of image stacks was performed by using Amira 5.6.0 (FEI Visualization Science Group, Burlington, USA) applying the “Volren” or “Voltex” function. Habitus photographs were taken by means of a Canon EOS 6D with the Canon MP-E 65 mm macro lens in a BK PLUS Lab system by Dun Inc. The flagella of *O. melanopus* and *O. duftschmidi* were photographed using the Direct to Sensor Microscope Lens with a 10× Mitutoyo objective mounted on a Canon EOS 7D camera in a BK PLUS Lab system by Dun Inc. Obtained images stacks were processed using Zerene Stacker and Adobe Photoshop CS6.

## Data repository

The set of TIFF-files from the microCT scan of the three (former) syntype specimens of *Oulema erichsonii* is deposited at the MLUH, that of *Oulema septentrionis* at ZMHU, copies are accessible through MorphDBase (https://www.morphdbase.de/ – OULEERIC_1, OULEERIC_2, OULESEPT_1).

## Results

### *Oulema septentrionis* (Weise, 1880)

**Figure 1.**
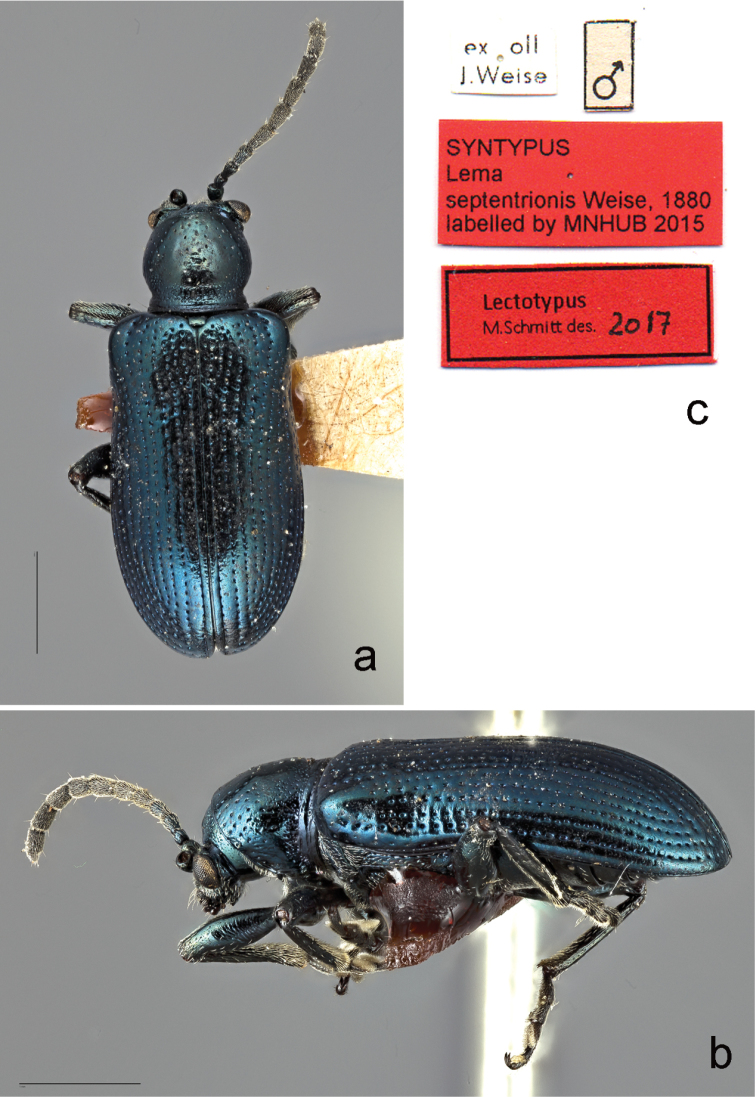
*
Oulema septentrionis* (Weise, 1880), lectotype. **a** dorsal **b** lateral from left **c** labels. Scale bars: 1 mm.

Of the 11 syntype specimens – all of them pinned - of *O. septentrionis* we scanned three specimens, among them one male that we designate here lectotype (Fig. 1 - male, pinned, Germany [with no locality information] / Weise, see Fig. 1c). Three of the paralectotypes have also no locality label. Nine specimens of the syntype series have the same red name label and an “ex coll. Weise label”as the lectotype (Fig. 1c). Of these, one has an additional handwritten label “septentrionis *”, one a small label “Müggel” (probably Müggelsee/lake Müggel in Berlin), one is labelled “Styria Ludg”, and one “Stettin”. The two remaining specimens have a simple label “Typus”, one of them additionally a label “Müggel” and a name label “Lema septentrionis Wse. / L.N. Medvedev det. 1973”. All paralectotypes were additionally labelled “Paralectotypus / M.Schmitt des. 2017”.

**Figure 2.**
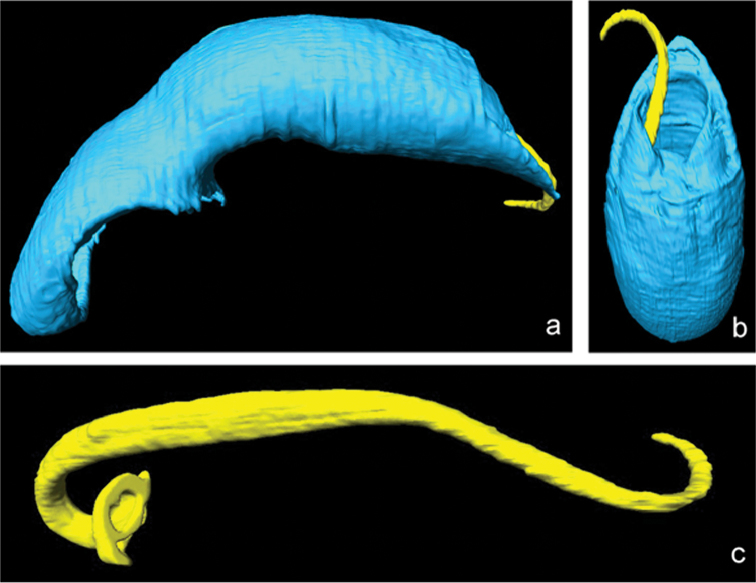
*
Oulema septentrionis*, lectotype; **a**: median lobe with flagellum lateral **b** apex of median lobe with flagellum dorsal; **c**: flagellum (not to scale). 3D reconstructed microCT-scans.

The 3D reconstruction revealed that the tip of the apex of the median lobe is pointing straight forward. Seen from the side, the apex of the median lobe has a wedge-like shape with upper and lower outline forming an angle of 40° (Fig. 2a). The ostium is oval and semicircular towards the tip of the apex, with the distal third of the flagellum laying outside the median lobe (Fig. 2b). The flagellum has a thicker portion basally and a thinner towards the tip, the latter being about as long as the basal part (Fig. 2c).

### *Oulema erichsonii* (Suffrian, 1841)

**Figure 3.**
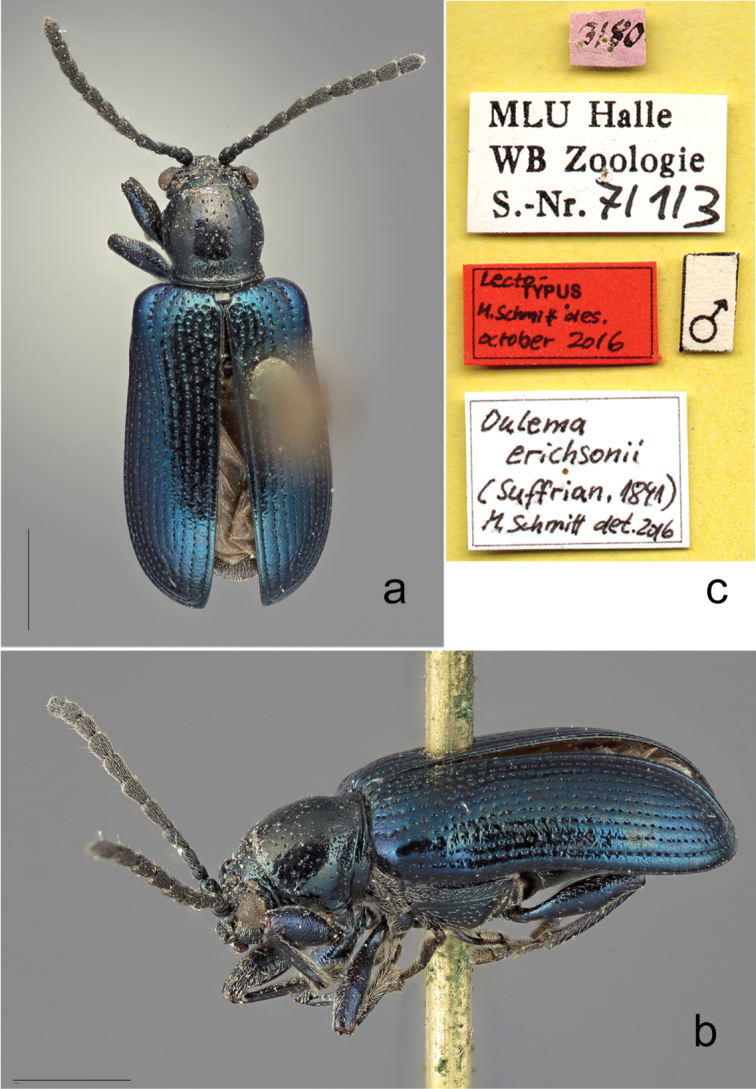
*
Oulema erichsonii* (Suffrian, 1841), lectotype. **a** dorsal **b** lateral from left **c** labels. Scale bars: 1 mm.

The scanned syntype of *O. erichsonii* specimens was a male. This we designated here lectotype (Fig. 3).

Material examined: The specimens do not bear a locality label but only tiny labels in different colours showing an individual number. In the book of records maintained by Christian Wilhelm Ludwig Eduard Suffrian (1805–1876), the localities for each numbered specimen are listed. These are:

**Lectotype** male, pinned, No. 3180 Elberfeld (Fig. 3), **Paralectotypes**, pinned: nos. 3178 & 3179 Kassel, no. 3181 Altenburg, no. 3174 Dortmund, all originally listed as “Lema cyanella”, nos. 4220, 4221, 4222, 4223 “Regio 1827”, probably meaning the environments of Aschersleben, where Suffrian lived and worked as a school teacher from 1825 until 1833 (Dohrn 1877). He entered nos. 4220-4223 as “Lema cyanella Gyl.” but explained in a note on the left margin of the page, obviously added later: “4220 ist eine neue Art, L. Erichsonii Mihi. Davon ist 4221.22. die var. β. mit schwarzem Halsstück, und 4123 var. γ. schwarz. Die Art ist durch Bau und Punktierung des Halsstücks, sowie durch den Bau der Flügeldecken hinreichend von L. cyanella Gyl. verschieden“ (4220 is a new species, L. Erichsonii mihi. Of these is 4221.22 the variety β with black thorax, 4123 var. γ black. The species is by shape and punctuation of the pronotum as well as by the shape of the elytra sufficiently different from L. cyanella Gyl.). No. 9883 Siegen, no. 11012 “Wald von Montabaur”, no. 19596 Münster, no. 27523 Moskau. The fourteenth specimen, no. 10162 from Mainz, is clearly an *Oulema obscura* (Stephens, 1831), so we put a name label accordingly on the pin. “Lema cyanella Gyl.” refers to Gyllenhal’s *Insecta suecica* (1813: 638), where this name is used for *Oulema obscura* (Stephens, 1831). All paralectotypes were additionally labelled “Paralectotypus / M.Schmitt des. 2017”.

**Figure 4.**
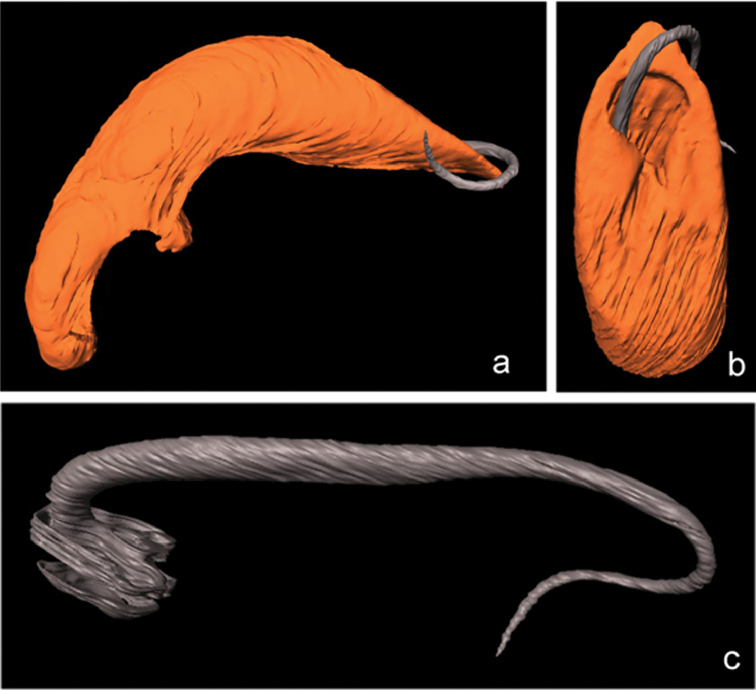
*
Oulema erichsonii*, lectotype; **a** median lobe with flagellum lateral **b** apex of median lobe with flagellum dorsal **c** flagellum (not to scale). 3D reconstructed microCT-scans.

The 3D reconstruction revealed that the tip of the apex of the median lobe is slightly bent downwards (“ventrally”). Seen from the side, the apex of the median lobe has a wedge-like shape with upper and lower outline forming an angle of 30° (Fig. 4a). The ostium is broad and semicircular towards the tip of the apex, with the distal half of the flagellum laying outside the median lobe (Fig. 4b). The flagellum has a thicker portion basally and a thinner towards the tip, the latter being considerably longer than the basal part (Fig. 4c).

### *Oulema melanopus* (Linnaeus, 1758) and *Oulema duftschmidi* (Redtenbacher, 1874)

We compared the flagella of the above species with those of the species of which they were suspected melanic forms. One male of *O. melanopus*: GERM. RHEINL. / UNKEL / 29.93.92 SIEDE // BACHTÄLCHEN / KAHLSCHLAG / UNT. VERBASCUM // LEMA MELANOPUS (L.) S.STR. / SIEDE DET. 92. One male of *O. duftschmidi*: same data as before, but LEMA DUFT- / SCHMIDI REDT. / SIEDE DET. 92.

**Figure 5.**
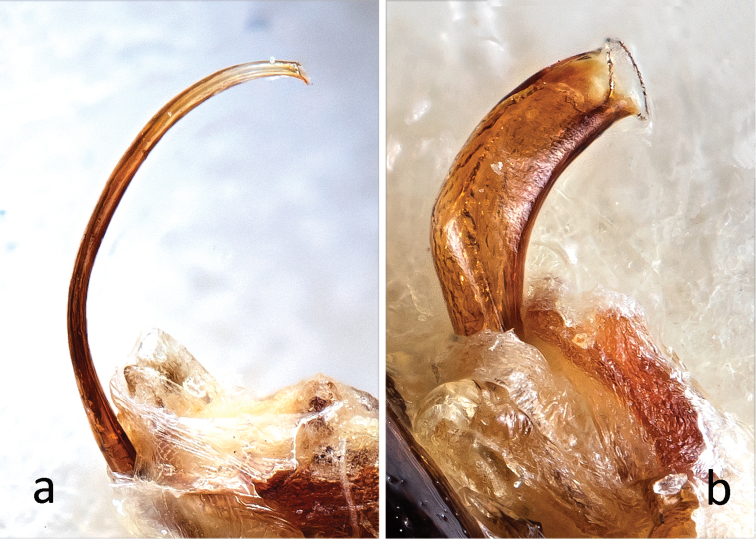
Flagella of **a** *Oulema duftschmidi* **b** *O. melanopus*, photos taken at the same magnification.

The flagella differ clearly from each other and from those of *O. erichsonii* and *O. septentrionis*. The flagellum of *O. melanopus* is short, stout, and only slightly curved, with a proportion of length/diameter=4.8. The flagellum of *O. duftschmidi* is long, slender, nearly semi-circular, without an inflexion point, and ca. 40 times longer than wide. Since the rim of the distal opening is complete, it is certain that the tips are not broken (Fig. 5).

## Discussion

Our study shows that *Oulema septentrionis* is not a melanic form of *Oulema melanopus*. The specimens from Ireland and Normandy identified as *Oulema septentrionis* by the authors of the website www.coleoptera.org.uk must belong to a different species, provided that the shape of the flagella differ between the supposed *O. septentrionis* and the specimens we investigated.

The outer morphology of *Oulema melanopus* (Linnaeus, 1758) and of *O. duftschmidi* (Redtenbacher, 1874) is extremely similar. Until Nicole Berti’s thorough investigation (1989) taxonomists treated the two forms as conspecific. Therefore, we used dissected specimens of both species for comparison with *O. septentrionis* and *O. erichsonii* (Fig. 5). The morphological comparison shows clearly that neither *O. septentrionis* nor *O. erichsonii* is conspecific with *O. melanopus* or *O. duftschmidi*.

The morphological differences in the aedeagus of the lectotypes of *Oulema septentrionis* and *O. erichsonii* concur with the differences in external morphology found in the literature (e.g., Weise 1893, Mohr 1966, Warchałowski 2003): *O. erichsonii* has a very finely punctate pronotum, whereas in *O. septentrionis* only the hind part of the pronotum is finely and deeply punctate. However, the elytral punctures appear very much the same, in contrast to the description in Mohr (1966). Altogether, the differences indicate that the two forms are separate morphospecies, which is also supported by Bukejs’ (2010) study on their aedeagi. Since we have no information on whether these morphospecies interbreed, and if so, with viable offspring, it is uncertain if they are biospecies. Both species are repeatedly mentioned as feeding on *Nasturtium*, which goes back to Weise (1893). Still, there are no confirmations of this statement nor did we find the species when inspecting *Nasturtium* in the field. In conclusion, we cautiously treat *Oulema septentrionis* and *O. erichsonii* for taxonomic purposes as different species based on our morphological investigation.

Our study corroborates that Micro-computed X-ray tomography (µCT) can be used successfully for non-invasive, non-destructive investigation of internal structures of dried beetle specimens, e.g. old type material, as was e.g. demonstrated for Lepidoptera (Simonsen and Kitching 2014) and damselflies (Steinhoff and Uhl 2015). A pixel size of 5.36 µm is sufficient to reveal the details necessary for a taxonomic analysis of the male copulatory apparatus in resting posture inside the specimen.
